# Dicots take a CYP from the herbicide detoxification cup

**DOI:** 10.1093/jxb/eraf138

**Published:** 2025-07-02

**Authors:** Danica Goggin

**Affiliations:** Australian Herbicide Resistance Initiative, UWA School of Agriculture and Environment, The University of Western Australia, Crawley, WA 6009 Australia

**Keywords:** *Amaranthus*, CYP72A1182, cytochrome P450, herbicide resistance, HPPD inhibitors, metabolism

## Abstract

This article comments on:

**Rigon CAG, Küpper A, Sparks C, Montgomery J, Peter F, Schepp S, Perez-Jones A, Tranel PJ, Beffa R, Dayan FE, Gaines TA.** 2025. Function of cytochrome P450 *CYP72A1182* in metabolic herbicide resistance evolution in *Amaranthus palmeri* populations. Journal of Experimental Botany **76**, 2891–2907 https://doi.org/10.1093/jxb/eraf114

This article comments on:

**Rigon CAG, Küpper A, Sparks C, Montgomery J, Peter F, Schepp S, Perez-Jones A, Tranel PJ, Beffa R, Dayan FE, Gaines TA.** 2025. Function of cytochrome P450 *CYP72A1182* in metabolic herbicide resistance evolution in *Amaranthus palmeri* populations. Journal of Experimental Botany **76**, 2891–2907 https://doi.org/10.1093/jxb/eraf114

**Food production is impeded by herbicide resistance in weeds in the same way that human health is affected by antibiotic resistance. The cytochrome P450 superfamily of enzymes (CYPs) plays a primary role in the metabolic detoxification of herbicides, but identification of the specific family members responsible for evolved resistance is challenging and has mainly been achieved in grass weeds. In a comprehensive study, Rigon *et al*. (2025) not only demonstrate the involvement of CYP72A1182 in detoxification of the herbicide tembotrione in a major dicot weed, but propose a molecular mechanism for its differential expression in resistant and susceptible plants.**

Efficient conventional food crop production is dependent on synthetic herbicides, as weed infestations can dramatically reduce crop yields. Repeated and intensive use of herbicides has resulted in the evolution of resistant weed populations across the world, and the economic cost runs into millions of US dollars per annum (Varah *et al*., 2024). Herbicide resistance in weeds generally results from mutations that alter target protein interactions or from mechanistic changes which minimize the amount of herbicide reaching the target; overwhelmingly, the latter involves metabolic modification of the herbicide molecule to a less phytotoxic form (Riechers *et al*., 2024). The first step of herbicide detoxification usually involves hydroxylation or dealkylation of the herbicide molecule to increase its polarity and reactivity, making it amenable to conjugation with polar molecules such as glutathione and/or sugars (Dimaano and Iwakami, 2020). Members of the cytochrome P450 (CYP) superfamily have long been implicated in these initial oxidative reactions (Yuan *et al*., 2007), although the difficulty in working with large numbers of candidate *CYP* genes (especially in sporadically annotated weed genomes) and their membrane-bound products means that relatively few studies have conclusively identified individual CYPs as being responsible for herbicide resistance. A recent phylogenetic analysis showed that all of the 30 CYPs (predominantly from crops and model plants) so far confirmed as either directly metabolizing herbicides or conferring resistance in transgenic organisms belong to two large clans, CYP71 and CYP72, whose members are involved in the biosynthesis of hormones and secondary metabolites (Casey and Dolan, 2023).

The evolution of herbicide resistance via enhanced metabolic detoxification in grass weeds and cereal crops dominates the literature, with the CYP81 subclass of the CYP71 clan as well as the CYP72 family playing a major role (Dimaano and Iwakami, 2020). However, more recent publications also describe CYP-mediated metabolic resistance in the highly damaging weeds in the dicot genus *Amaranthus*, many populations of which are resistant to multiple herbicide modes of action (Heap, 2025). Enhanced production of hydroxylated metabolites of the synthetic auxin 2,4-D (de Figueiredo *et al*., 2022), the triketones mesotrione (Ma *et al*., 2013) and tembotrione (Küpper *et al*., 2018), and the pyrazolone topramezone (Lygin *et al*., 2018) is associated with resistance in *Amaranthus* populations, with the involvement of CYPs being further implicated by the negative effect of applied CYP inhibitors on metabolite production. Based on *in vitro* enzyme assays, demethylation of the α-chloroacetamide *S*-metolachlor by resistant *A. tuberculatus* is also likely to be catalysed by a CYP (Strom *et al*., 2021). It is interesting to note that the herbicide group most commonly represented in the above list is the 4-hydroxyphenylpyruvate dioxygenase (HPPD) inhibitors (mesotrione, tembotrione, and tompramezone), which prevent a susceptible plant from synthesizing plastoquinone, leading to loss of photosynthetic and protective pigments and lipid-soluble antioxidants (see Ma and Guo, 2024, for a summary of HPPD inhibitors and their increasing importance in weed control). Furthermore, of the 16 cases of HPPD inhibitor resistance reported so far in *Amaranthus* spp. (Heap, 2025), a target site-based mechanism of resistance (increased *HPPD* expression) is proposed in only one (Nakka *et al*., 2017). This leaves a large number of non-target site-based cases of resistance, with their associated genes and proteins, to be identified so that HPPD inhibitor resistance in *Amaranthus* can be understood and mitigated.

## Pinning down the tembotrione detoxification enzyme

**Fig. 1.**
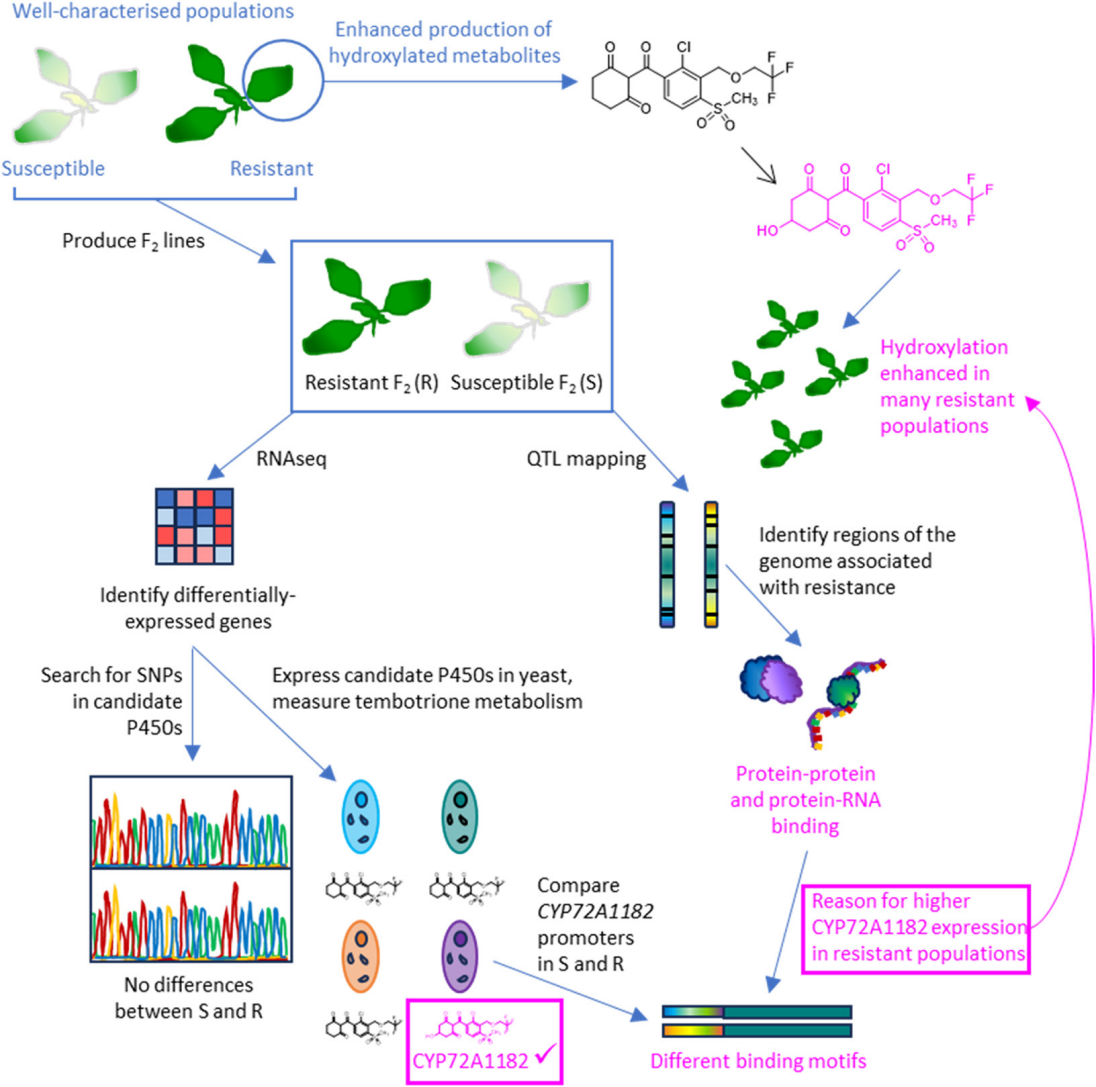
Summary of the approach taken to identify tembotrione-metabolizing enzymes in resistant *Amaranthus palmeri*. A parental resistant population with enhanced tembotrione hydroxylating activity was crossed with a susceptible population to produce pseudo-F_2_ lines segregating for tembotrione resistance. Candidate resistance genes were identified by RNA-seq and validated by measuring hydroxy-tembotrione production in yeast overexpressing each gene. Polymorphisms in the promoter of the validated gene, *CYP72A1182*, plus a QTL analysis pinpointing genomic regions associated with resistance, suggest that differential transcriptional regulation of *CYP72A1182* is responsible for the differences in hydroxylating capacity between susceptible and resistant plants.

The increased tembotrione metabolites initially identified in resistant *A. palmeri* by Küpper *et al*. (2018) were subsequently shown to be hydroxy-tembotrione and its sugar-conjugated derivatives. Rigon *et al*. (2025) have now described a remarkable effort to identify the hydroxylating enzyme involved. In a multi-pronged approach (summarized in Fig. 1), the authors started by pair-crossing their tembotrione-susceptible and -resistant populations, producing pseudo-F_2_ lines of uniform genetic background that segregated into individuals susceptible (S) or resistant (R) to tembotrione. The pseudo-F_2_ lines were then used in two studies. Firstly, an analysis of gene expression was performed using RNA-seq to identify genes that were differentially expressed in S and R individuals, either constitutively or in response to tembotrione treatment. Secondly, regions of the genome associated with tembotrione resistance were identified by quantitative trait locus (QTL) mapping, so that the search for resistance-conferring genes could be narrowed down. The RNA-seq study revealed (as they generally do) a considerable number of differentially expressed genes. However, knowing that hydroxylation/conjugation was the mechanism of resistance in the R plants, Rigon *et al*. (2025) focused on the gene families associated with xenobiotic detoxification. They pinpointed four *CYP* genes as candidate resistance genes, along with several genes encoding conjugating enzymes, and two transcription factors whose importance will soon become apparent. This is the point, before the difficulties of functional characterization, at which many studies using transcriptomics to identify candidate resistance genes come to an end.

Rigon *et al*. (2025) then transformed yeast cells with their four candidate *CYP* genes in order to measure the ability of the transgenic yeast to hydroxylate tembotrione. Only one of the encoded P450s, named CYP72A1182 (a member of the known herbicide-metabolizing CYP72 clan: Casey and Dolan, 2023) was able to produce the hydroxylated tembotrione derivatives identified by Küpper *et al*. (2018). Sequencing of *CYP72A1182* in S and R plants revealed no resistance-related polymorphisms in the coding sequence, but the promoter region contained insertions that changed the binding-site motifs. Interestingly, those insertions unique to the R plants were sites for the binding of abiotic stress-responsive transcription factors (Rigon *et al*., 2025). Meanwhile, the genetic mapping study revealed a major QTL on chromosome 4 whose genes, whilst not including *CYP72A1182*, function in protein–protein and protein–nucleic acid binding. This finding, combined with the higher expression of two transcription factors in the R plants and the different binding motifs in the *CYP72A1182* promoter, led the authors to hypothesize that the enhanced ability of R plants to hydroxylate tembotrione via CYP72A1182 is linked to a set of regulatory genes that increase the expression of *CYP72A1182* on both a constitutive and a tembotrione-responsive basis. Screening of another 10 putative-resistant populations of *A. palmeri* revealed that the level of *CYP72A1182* expression and hydroxy-tembotrione production was largely correlated with the level of tembotrione resistance, confirming the important role of CYP72A1182-mediated tembotrione hydroxylation in the resistance phenotype (Rigon *et al*. 2025). The only study so far delving rigorously into the regulation of genes conferring non-target site-based herbicide resistance showed, in the multiple-resistant grass weed *Echinochloa phyllopogon*, that two catalytically promiscuous CYP81s (clan CYP71) and a more selective CYP709 (clan CYP72) were transcriptionally linked and potentially regulated by a single genetic element (Suda *et al*., 2023). It would be very interesting to use *Amaranthus* to investigate if a ‘master switch’ also exists in multiple-resistant dicot weeds, which have until now lagged behind the grasses in the race for validated CYP-mediated herbicide detoxification.

## Putting weedy *Amaranthus* to work

Detailed knowledge of how resistant weed populations can detoxify herbicides is essential for the development of mitigation strategies, in both the short term (via tailoring of existing weed management techniques to the specific resistance case) and the long term (via biorational design of ‘unmetabolizable’ herbicides and the development of new transgenic crops with herbicide tolerance). Rigon *et al*. (2025) have shown that *Amaranthus* can be used as an informative dicot species to investigate the role of cytochrome P450 in HPPD inhibitor detoxification at both the enzymatic and the regulatory level. Given that non-target site-based mechanisms seem to be the preferred evolutionary pathway for HPPD inhibitor resistance, and this mode of action is so important in *Amaranthus* control (inevitably resulting in the selection of a great many resistant populations), the work of Rigon *et al*. (2025) could be used as a springboard to identify not only other CYPs, but whole other enzyme families potentially involved in the first step of HPPD-inhibiting herbicide detoxification. An unusual pathway of ketone reduction of an uncommercialized HPPD inhibitor was identified in a resistant *Amaranthus* population (Concepcion *et al*., 2021), but the enzyme involved was not identified. Similarly, it is unknown if *Amaranthus* has the potential to hydroxylate triketone-class HPPD inhibitors via Fe(II)/2-oxoglutarate-dependent oxygenase activity, as has been observed in rice (Maeda *et al*., 2019). With the additional questions of how the expression of these detoxification enzymes is regulated, and if a ‘master’ gene regulates a cohort of related or unrelated enzymes, it is clear that there is a whole world of evolution still to explore in our agricultural fields.

## References

[CIT0001] Casey A , Dolan L. 2023. Genes encoding cytochrome P450 monooxygenases and glutathione S-transferases associated with herbicide resistance evolved before the origin of land plants. PLoS One 18, e0273594. doi: https://doi.org/10.1371/journal.pone.0273594. 36800395 PMC9937507

[CIT0002] Concepcion JCT , Kaundun SS, Morris JA, Hutchings S-J, Strom SA, Lygin AV, Riechers DE. 2021. Resistance to a nonselective 4-hydroxyphenylpyruvate dioxygenase-inhibiting herbicide via novel reduction–dehydration–glutathione conjugation in *Amaranthus tuberculatus*. New Phytologist 232, 2089–2105. doi: https://doi.org/10.1111/nph.17708. 34480751 PMC9292532

[CIT0003] de Figueiredo MRA , Barnes H, Boot CM, de Figueiredo ABTB, Nissen SJ, Dayan FE, Gaines TA. 2022. Identification of a novel 2,4-D metabolic detoxification pathway in 2,4-D-resistant waterhemp (*Amaranthus tuberculatus*). Journal of Agricultural and Food Chemistry 70, 15380–15389. doi: https://doi.org/10.1021/acs.jafc.2c05908. 36453610

[CIT0004] Dimaano NG , Iwakami S. 2020. Cytochrome P450-mediated herbicide metabolism in plants: current understanding and prospects. Pest Management Science 77, 22–32. doi: https://doi.org/10.1002/ps.6040. 32776423

[CIT0005] Heap I. 2025. The international survey of herbicide resistant weeds. www.weedscience.org. Accessed 17 January 2025.

[CIT0006] Küpper A , Peter F, Zöllner P, Lorentz L, Tranel PJ, Beffa R, Gaines TA. 2018. Tembotrione detoxification in 4-hydroxyphenylpyruvate dioxygenase (HPPD) inhibitor-resistant Palmer amaranth (*Amaranthus palmeri* S. Wats.). Pest Management Science 74, 2325–2334. doi: https://doi.org/10.1002/ps.4786. 29105299

[CIT0007] Lygin AV , Kaundun SS, Morris JA, Mcindoe E, Hamilton AR, Riechers DE. 2018. Metabolic pathway of topramezone in multiple-resistant waterhemp (*Amaranthus tuberculatus*) differs from naturally tolerant maize. Frontiers in Plant Science 9, 1644. doi: https://doi.org/10.3389/fpls.2018.01644. 30519248 PMC6258821

[CIT0008] Ma L , Guo Y. 2024. Overcoming weeds: breeding herbicide-resistant crops via directed evolution. Journal of Experimental Botany 75, 6889–6892. doi: https://doi.org/10.1093/jxb/erae445. 39656673 PMC11630303

[CIT0009] Ma R , Kaundun SS, Tranel PJ, Riggins CW, McGinness DL, Hager AG, Hawkes T, McIndoe E, Riechers DE. 2013. Distinct detoxification mechanisms confer resistance to mesotrione and atrazine in a population of waterhemp. Plant Physiology 163, 363–377. doi: https://doi.org/10.1104/pp.113.223156. 23872617 PMC3762656

[CIT0010] Maeda H , Murata K, Sakuma N, et al 2019. A rice gene that confers broad-spectrum resistance to β-triketone herbicides. Science 365, 393–396. doi: https://doi.org/10.1126/science.aax0379. 31346065

[CIT0011] Nakka S , Godar AS, Wani PS, Thompson CR, Peterson DE, Roelofs J, Jugulam M. 2017. Physiological and molecular characterization of hydroxyphenylpyruvate dioxygenase (HPPD)-inhibitor resistance in Palmer amaranth (*Amaranthus palmeri* S. Wats.). Frontiers in Plant Science 8, 555. doi: https://doi.org/10.3389/fpls.2017.00555. 28443128 PMC5387043

[CIT0012] Riechers DE , Soltani N, Chauhan BS, Concepcion JCT, Geddes CM, Jugulam M, Kaundun SS, Preston C, Wuerrfel J, Sikkema PH. 2024. Herbicide resistance is complex: a global review of cross-resistance in weeds within herbicide groups. Weed Science 72, 465–486. doi: https://doi.org/10.1017/wsc.2024.33.

[CIT0013] Rigon CAG , Küpper A, Sparks C, et al 2025. Function of cytochrome P450 *CYP72A1182* in metabolic herbicide resistance evolution in Amaranthus palmeri populations. Journal of Experimental Botany 76, 2891–2907. 10.1093/jxb/eraf114 PMC12223498 40067326

[CIT0014] Strom SA , Hager AG, Concepcion JCT, Seiter NJ, Davis AS, Morris JA, Kaundun SS, Riechers DE. 2021. Metabolic pathways for *S*-metolachlor detoxification differ between tolerant corn and multiple-resistant waterhemp. Plant and Cell Physiology 62, 1770–1785. doi: https://doi.org/10.1093/pcp/pcab132. 34453831 PMC8664635

[CIT0015] Suda H , Kubo T, Yoshimoto Y, et al 2023. Transcriptionally linked simultaneous overexpression of P450 genes for broad-spectrum herbicide resistance. Plant Physiology 192, 3017–3029. doi: https://doi.org/10.1093/plphys/kiad286. 37195199 PMC10400030

[CIT0016] Varah A , Ahodo K, Childs DZ, et al 2024. Acting pre-emptively reduces the long-term costs of managing herbicide resistance. Scientific Reports 14, 6201. doi: https://doi.org/10.1038/s41598-024-56525-0. 38485959 PMC10940647

[CIT0017] Yuan JS , Tranel PJ, Stewart CN Jr. 2007. Non-target-site herbicide resistance: a family business. Trends in Plant Science 12, 6–13. doi: https://doi.org/10.1016/j.tplants.2006.11.001. 17161644

